# Apoptosis of human gastric cancer SGC-7901 cells induced by podophyllotoxin

**DOI:** 10.3892/etm.2014.1606

**Published:** 2014-03-06

**Authors:** CHEN-FENG JI, YU-BIN JI

**Affiliations:** 1 Engineering Research Center of Natural Anticancer Drugs, Ministry of Education, Harbin University of Commerce, Harbin, Heilongjiang 150076, P.R. China; 2 Center of Research on Life Science and Environmental Science, Harbin University of Commerce, Harbin, Heilongjiang 150076, P.R. China

**Keywords:** human gastric cancer, podophyllotoxin, apoptosis, mechanism

## Abstract

Numerous studies have demonstrated that podophyllotoxin and its derivatives exhibit antitumor effects. The aim of the present study was to investigate SGC-7901 cell apoptosis and the underlying mechanism induced by podophyllotoxin. SGC-7901 cells were treated with varying concentrations of podophyllotoxin. MTT assays and flow cytometry were used to evaluate the effects of podophyllotoxin on the proliferation and apoptosis of SGC-7901 cells, while fluorescence inverted microscopy was used to observe the morphology of SGC-7901 cells that had been dyed with Hoechst 33258. In addition, laser scanning confocal microscopy was used to analyze the mitochondrial membrane potential (MMP) of SGC-7901 cells dyed with Rhodamine 123. Western blotting was performed to analyze the expression levels of cytochrome *c* (cyt-*c*), caspase-9 and caspase-3 in the SGC-7901 cells. The results indicated that podophyllotoxin was capable of inhibiting growth and inducing the apoptosis of SGC-7901 cells in a dose-dependent manner, causing cell cycle arrest at the G_2_/M phase. After 48 h of treatment, the apoptotic morphology of SGC-7901 cells was clear, exhibiting cell protuberance, concentrated cytoplasms and apoptotic bodies. Following 24 h of treatment, the MMP of the SGC-7901 cells decreased. In addition, after 48 h, the expression of cyt-*c* was shown to be upregulated, while the expression levels of pro-caspase-9 and pro-caspase-3 in the SGC-7901 cells were shown to be downregulated. In conclusion, apoptosis can be induced in SGC-7901 cells by podophyllotoxin, potentially via a mitochondrial pathway, indicating that podophyllotoxin may be a potent agent for cancer treatment.

## Introduction

Gastric cancer (GC) is one of the most common cancers worldwide, particularly in East Asia and East Europe. Furthermore, the incidence and mortality rates of GC are high, accounting for ~1,000,000 mortalities annually. Therefore, GC is a significant problem in terms of global health (1). Despite advances in surgery and chemotherapy for colon cancer, the outcomes of anticancer therapy remain unsatisfactory, thus, further improvements are required. A number of pharmacological experiments have demonstrated that numerous naturally active components, isolated from plants and herbs, exhibit antitumor effects and may be potent agents for cancer treatment (2,3).

Podophyllotoxin is a lignan extracted from the *Podophyllum* plant, with a molecular formula of C_22_H_22_O_8_ and a molecular weight of 414 Da. This compound and its derivatives have great significance as antineoplastic drugs and antiviral agents due to the biological activities that they exhibit. Podophyllotoxin and its derivatives are currently used in chemotherapy for various cancer types, including cervical carcinoma, osteosarcoma, nasopharyngeal carcinoma, colon cancer, breast cancer, prostate cancer, testicular carcinoma, small cell lung cancer and lymphoma (4–8). However, studies investigating the antitumor effect on GC are limited (9) and the molecular mechanism remains unclear. In the present study, the inhibition of cell growth and apoptosis induced by podophyllotoxin was investigated in the human GC SGC-7901 cell line, and the underlying mechanism was studied through the mitochondrial pathway.

## Materials and methods

### Reagents

Podophyllotoxin, MTT, propidium iodide (PI), Hoechst 33258 and Rodamine 123 were all purchased from Sigma-Aldrich (St. Louis, MO, USA). Hydroxycamptothecin (HCPT) was purchased from Harbin Shengtai Pharmaceutical Co., Ltd. (Harbin, China); RPMI 1640 culture medium was purchased from Thermo Fisher Scientific Inc. (Waltham, MA, USA); fetal bovine serum (FBS) was obtained from Hangzhou Sijiqing Biological Engineering Materials Co., Ltd. (Hangzhou, China) and trypsin was purchased from Gibco-BRL (Rockville, MD, USA). Additionally, mouse anti-human β-actin, cytochrome c (cyt-c), caspase-9 and caspase-3 polyclonal antibodies, alkaline phosphatase (AP)-conjugated goat anti mouse polyclonal antibody, SDS-PAGE sample loading buffer, blocking buffer, TBST, buffer5-bromo-4-chloro-3-indolyl-phosphate (BCIP)/nitroblue tetrazolium (NBT) alkaline phosphatase color development kit (Beyotime Institute of Biotechnology, Haimen, China); detergent-compatible protein assay kit (Bio-Rad, Hercules, CA, USA).

### Apparatus

The CKX 41 inverted fluorescence microscope was purchased from Olympus (Tokyo, Japan) and the mini electrophoresis meter and microplate reader were purchased from Bio-Rad (Hercules, CA, USA). The EPICS XL flow cytometer was purchased from Beckman Coulter (Brea, CA, USA); the SP2 laser confocal scanning microscope was purchased from Leica (Solms, Germany) and the CO-150 CO_2_ incubator was purchased from New Brunswick Scientific (Edison, NJ, USA).

### Cell culture

The human GC SGC-7901 cell line was provided by the Center of Research and Development on Life Sciences and Environmental Sciences of Harbin University of Commerce (Harbin, China). SGC-7901 cells were grown in RPMI 1640 culture medium containing 10% heat-inactivated FBS at 37°C in a humidified atmosphere of 5% CO_2_.

### Antitumor activity of podophyllotoxin on SGC-7901 cells

Exponentially growing cells were washed, digested with trypsin and suspended in RPMI 1640 medium until a concentration of 1×10^5^ cells/ml was achieved. In a 96-well plate, 100 μl cell suspension per well was cultured for 24 h. The cells were subsequently incubated with varying concentrations of podophyllotoxin for a further 72 h; 12 wells were used per concentration. Cells incubated with only 100 μl RPMI 1640 culture medium served as a control group. MTT was dissolved in phosphate-buffered saline (PBS) to provide a solution with a final concentration of 0.5 mg/ml. After 72 h, the cell suspension was discarded and 200 μl MTT solution (0.5 mg/ml) was added to each well and incubated at 37°C for 4 h. All media were removed and 150 μl dimethyl sulfoxide was added to each well in order to dissolve the purple formazan crystals. The plate was agitated for 3 min and the spectrophotometric absorbance at 570 nm was measured using a microplate reader. The inhibitory rate and IC_50_ was calculated.

### Effect of podophyllotoxin on cell cycle and apoptosis of SGC-7901 cells

Cells (5×10^4^/ml; 1 ml) were planted in 24-well plates and cultured for 24 h with three wells per group. Subsequently, the cells were treated with various drug concentrations for 48 h. The cells were harvested via trypsinization, washed twice with cold PBS and fixed in 70% cold ethanol for 12 h at 4°C. The fixation fluid was then discarded and the cells were washed with PBS twice. PI was added to the samples for 30 min for staining and the samples were measured by flow cytometry with an excitation wavelength of 488 nm.

### Effect of podophyllotoxin on the morphology of SGC-7901 cells

Cells (5×10^4^/ml; 1 ml) were planted in 6-well plates, cultured for 24 h and treated with varying drug concentrations for 48 h. Cells were harvested via trypsinization, washed twice with cold PBS and fixed in 4% paraformaldehyde for 30 min at 4°C. The fixation fluid was then discarded and the cells were washed with PBS twice. Hoechst 33258 was added for 20 min, after which the dye was discarded and the cells were washed twice with PBS. The cells were observed using an inverted fluorescence microscope.

### Effect of podophyllotoxin on the mitochondrial membrane potential (MMP) in SGC-7901 cells

Cells (5×10^4^/ml; 1 ml) were planted in 6-well plates, cultured for 24 h and treated with various drug concentrations for 24 h. Cells were harvested via trypsinization, washed twice with cold PBS and loaded with Rhodamine 123 (5 μg/ml) for 30 min at 37°C. The fluorescence intensity of the MMP was observed using laser scanning confocal microscopy.

### Effect of podophyllotoxin on the expression of cyt-c, pro-caspase-9 and pro-caspase-3 in SGC-7901 cells

Cells (5×10^4^/ml; 1 ml) were planted in 6-well-plates, cultured for 24 h and subsequently treated with varying concentrations of podophyllotoxin for 48 h. Cytoplasm extracts were prepared with 150 μl cell lysis buffer on ice for 30 min. Following centrifugation at 10,000 × g at 4°C for 10 min, the supernatant was collected and the protein concentration was quantified using the detergent-compatible protein assay kit. Proteins were mixed with SDS-PAGE sample loading buffer (Beyotime Institute of Biotechnology). In total, a 40-μg sample of protein was separated in a 10% polyacrylamide gel and blotted on a nitrocellulose membrane. The blots were blocked with blocking buffer for 2 h at room temperature and incubated with anti-cyt-*c*, anti-caspase-9 and anti-caspase-3 antibodies for 12 h at 4°C. Subsequently, the nitrocellulose membranes were washed with TBST buffer three times and incubated with AP-conjugated goat anti-mouse antibody in blocking buffer for 2 h at R.T. Then the nitrocellulose membranes were washed with TBST buffer three times and detected by BCIP/NBT alkaline phosphatase color development kit (Beyotime Institute of Biotechnology). The bands were then visualized and quantified using the Gel Doc XR imaging system (Bio-Rad, Hercules, CA, USA).

### Statistical analysis

Data are expressed as the mean ± SD. Statistical analyses were performed using analysis of variance to compare the various groups. SPSS 15.0 software was used for statistical analysis (SPSS, Inc., Chicago, IL, USA). P<0.05 was considered to indicate a statistically significant difference.

## Results

### Antitumor activity of podophyllotoxin on SGC-7901 cells

**Figure 1:**
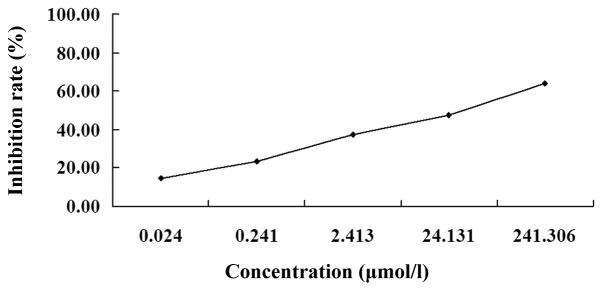
Inhibitory rate of podophyllotoxin on SGC-7901 cells.

The MTT assay revealed that following treatment with various concentrations of podophyllotoxin for 72 h, podophyllotoxin exhibited an inhibitory effect on the proliferation of SGC-7901 cells in a concentration-dependent manner. The optical density values of the treated groups exhibited statistically significant differences when compared with the control group (P<0.01). The IC_50_ was 26.30 μmol/l (Fig. 1).

### Effect of podophyllotoxin on the cell cycle and apoptosis of SGC-7901 cells

**Figure 2:**
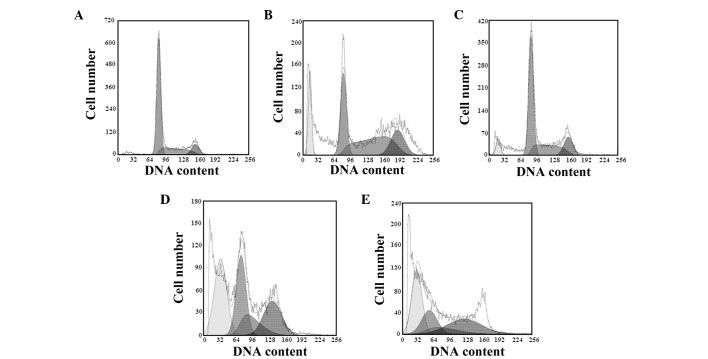
Effects of podophyllotoxin on the apoptosis of SGC-7901 cells treated with (A) negative control, (B) 13.15 μmol/l podophyllotoxin, (C) 26.30 μmol/l podophyllotoxin, (D) 52.60 μmol/l podophyllotoxin and (E) 55 μmol/l HCPT as a positive control. HCPT, hydroxycamptothecin.

Following treatment with varying concentrations of podophyllotoxin for 48 h, the cell cycle changed and the number of cells in the G_0_/G_1_ and S phases decreased, while the number of cells in the G_2_/M phase increased. The apoptotic peaks appeared gradually in a concentration-dependent manner, and the apoptosis rates were 11.06, 20.39 and 33.67% in the 13.15, 26.30 and 52.60 μmol/l podophyllotoxin groups, respectively, as shown in Table I and Fig. 2.

### Effect of podophyllotoxin on the morphology of SGC-7901 cells

**Figure 3:**
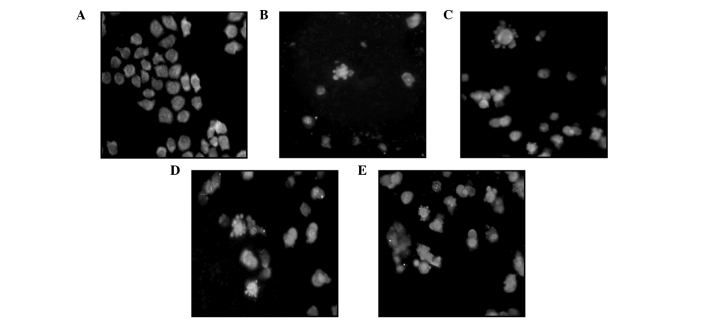
Effect of podophyllotoxin on the morphology of SGC-7901 cells treated with (A) negative control, (B) 13.15 μmol/l podophyllotoxin, (C) 26.30 μmol/l podophyllotoxin, (D) 52.60 μmol/l podophyllotoxin and (E) 55 μmol/l HCPT as a positive control. HCPT, hydroxycamptothecin.

Under an inverted fluorescence microscope, the cells in the control group were observed to grow normally and the fluorescence in the cell nuclei was uniform. Following treatment with various concentrations of podophyllotoxin for 48 h, a high proportion of cells exhibited apoptosis-like changes, including cell detachment, cytoplasmic condensation and the appearance of apoptotic bodies, in a concentration-dependent manner (Fig. 3).

### Effect of podophyllotoxin on the MMP in SGC-7901 cells

**Figure 4:**
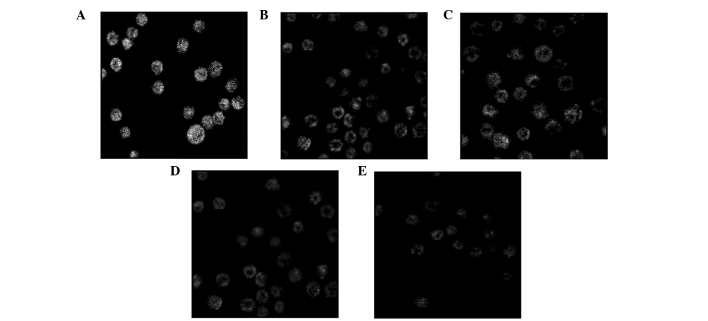
Effect of podophyllotoxin on the MMP in SGC-7901 cells treated with (A) negative control, (B) 13.15 μmol/l podophyllotoxin, (C) 26.30 μmol/l podophyllotoxin, (D) 52.60 μmol/l podophyllotoxin and (E) 55 μmol/l HCPT as a positive control. MMP, inner mitochondrial membrane potential; HCPT, hydroxycamptothecin.

Following treatment with various concentrations of podophyllotoxin for 24 h, the fluorescence intensity of Rhodamine 123 in cells decreased, indicating that the level of the MMP had decreased. With increasing drug concentrations, the level of the MMP decreased accordingly and statistically significant differences were observed when compared with the control group (P<0.05), as shown in Table II and Fig. 4.

### Effect of podophyllotoxin on the expression levels of cyt-c, pro-caspase-9, pro-caspase-3 and caspase-3 in SGC-7901 cells

**Figure 5:**
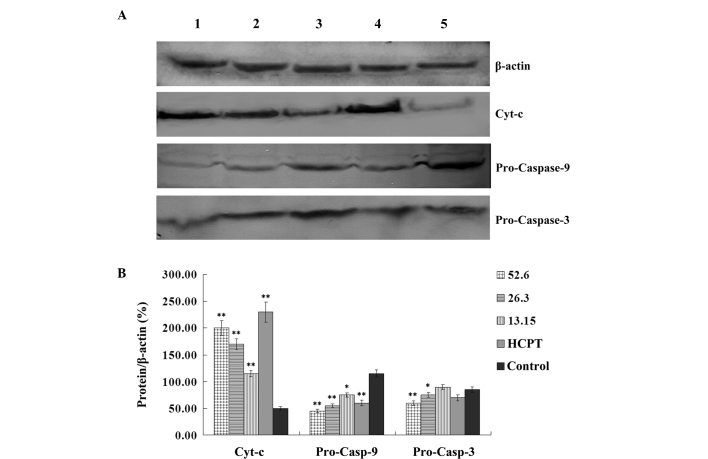
(A) Representative blot and (B) quantitative results demonstrating the effect of podophyllotoxin on apoptosis-related proteins in SGC-7901 cells treated with: Lane 1, 52.60 μmol/l podophyllotoxin; lane 2, 26.30 μmol/l podophyllotoxin; lane 3, 13.15 μmol/l podophyllotoxin; lane 4, 55 μmol/l HCPT (positive control); and lane 5, negative control. HCPT, hydroxycamptothecin.

Following treatment with various concentrations of podophyllotoxin for 24 h, the expression levels of apoptosis-related proteins in SGC-7901 cells changed. The expression level of cyt-*c* increased, while the expression levels of pro-caspase-9 and pro-caspase-3 decreased in a concentration-dependent manner. Statistically significant differences were observed when compared with the control group (P<0.05), as shown in Fig. 5.

## Discussion

Apoptosis is a rigorous, active and orderly process of cell death that is regulated by numerous genes in order to maintain the stability of the intracellular environment. The mitochondrial pathway is one of three major apoptotic pathways. This pathway involves the induction of apoptosis factors, the life-or-death apoptosis switch, the start molecules of apoptosis (cyt-c) and the effector molecules of apoptosis, including caspase-9 and caspase-3.

A change in the permeability of the mitochondrial membrane is the most significant event in apoptosis and this permeability is controlled by the mitochondrial permeability transition pore (MPTP), which is the key to the apoptotic mitochondrial pathway (10). The MMP is a result of the asymmetric distribution of protons and other ions across the inner membrane of the mitochondrion, and is necessary for sustaining mitochondrion function. The MMP is one of the best indicators of mitochondrion permeability (11). In the present study, the fluorescent probe Rhodamine 123 was used for staining. The experimental results demonstrated that following the treatment of SGC-7901 cells with podophyllotoxin for 24 h, the MPTP channels opened, the MMP decreased and the permeability of the mitochondrial membrane increased, resulting in irreversible cell apoptosis.

The release of cyt-*c* occurs in the early stages of cell apoptosis (12). Cyt-*c* is released into the cytoplasm through mitochondrial outer membrane permeabilization, which is regulated by MPTP or members of the Bcl-2 family. The release of cyt-*c* results in the activation of caspases. Caspases are a type of proenzyme that, under normal conditions, contain no reactive site. In caspase-dependent mitochondrial pathways, cyt-*c* is released from the mitochondria and interacts with aminophospholipid transferase and apoptotic peptidase activating factor 1, subsequently converting these molecules to polymers and promoting their interaction with caspase-9 to form apoptotic bodies (13). Cyt-*c* is capable of activating caspase-9 by hydrolyzing its proenzyme, and the activated caspase-9 further activates caspase-3, leading to cell apoptosis (14). The results of the present study demonstrate that podophyllotoxin facilitates the release of cyt-*c* from the mitochondrion into the cytoplasm, which decreases the expression levels of pro-caspase-9 and pro-caspase-3, leading to the caspase cascade with the formation of a caspase-dependent pathway. Therefore, apoptosis is induced in SGC-7901 cells via the mitochondrial pathway.

In conclusion, podophyllotoxin induces apoptosis in the human GC SGC-7901 cell line through the mitochondrial pathway.

## Figures and Tables

**Table I:** Effect of podophyllotoxin on the cell cycle of SGC-7901 cells (3 wells).

		Cell cycle (%)		
		
Group	Concentration (μmol/l)	G_0_/G_1_	S	G_2_/M
Control	0.00	65.166±0.125	26.754±0.114	14.080±1.074
Podophyllotoxin	26.30	43.049±0.102^b^	23.526±0.081^a^	22.425±0.202^b^
	52.60	34.737±0.082^b^	18.004±0.142^b^	27.259±0.736^b^
HCPT	55.00	32.210±0.043^b^	45.137±1.168^b^	22.653±0.086^b^
	13.15	55.351±0.096^a^	24.464±0.107	18.185±0.671^a^

Values are expressed as the mean±SD.

a P<0.05, vs. control;

b P<0.01, vs. control.

HCPT, hydroxycamptothecin.

**Table II:** Effect of podophyllotoxin on MMP in SGC-7901 cells (20 wells).

Group	Concentration (μmol/l)	Mean fluorescence intensity
Control	0.00	64.13±4.22
Podophyllotoxin	26.30	28.37±7.85^b^
	52.60	20.17±4.06^b^
HCPT	55.00	27.55±9.16^b^
	13.15	44.80±1.97^b^

Values are expressed as the mean±SD.

a P<0.05, vs. control.

b P<0.01, vs. control.

HCPT, hydroxycamptothecin; MMP, inner mitochondrial membrane potential.
